# Video encryption/compression using compressive coded rotating mirror camera

**DOI:** 10.1038/s41598-021-02520-8

**Published:** 2021-11-30

**Authors:** Amir Matin, Xu Wang

**Affiliations:** Institute of Physics and Quantum Science, Heriot Watt University, Third Gait, Currie, Edinburgh, EH14 4AS UK

**Keywords:** Electrical and electronic engineering, Imaging and sensing, Transmission light microscopy, Imaging techniques

## Abstract

Compressive coded rotating mirror (CCRM) camera is a novel high-speed imaging system that operates under amplitude optical encoding and frame sweeping modalities in a passive imaging mode that is capable of reconstructing 1400 frames from a single shot image acquisition and achieves the highest compression ratio of 368 compared to the other compressive sensing (CS) based single-shot imaging modalities. The integrated optical encoding and compression adds a strong layer of encryption on the observed data and facilitates the integration of the CCRM camera with the imaging applications that require highly efficient data encryption and compression due to capturing highly sensitive data or limited transmission and storage capacities. CCRM uses amplitude encoding that significantly extends the key space where the probability of having the exact encoder pattern is estimated as $P\left( A\right) \ =\ 1/{10}^{122,500}$, hence drastically reducing the possibility of data recovery in a brute force manner. Data reconstruction is achieved under CS based algorithms where the obtained amplitude-based pattern from optical encoder operates as the key in the recovery process. Reconstruction on the experimental as well as the synthetic data at various compression ratios demonstrate that the estimated key with less than 95$\%$ matching elements were unable to recover the data where the achieved averaged structural similarity (SSIM) of 0.25 before 95$\%$ encoder similarity and 0.85 SSIM at 100$\%$ encoder similarity demonstrates the high-sensitivity of the proposed optical encryption technique.

## Introduction

With recent advancements in high-speed imaging technologies, especially with the introduction of compressive sensing into their operation principle, high-speed imaging systems have become accessible for a wider range of applications, from capturing natural and ordinary dynamic scenes^1,2^ to high-throughput cell screening and classification^3–5^. These imaging systems however were associated with their unique disadvantages such as the requirement for expensive short-pulse laser (operation only in active mode) and the dependency on the precise repetition of the ultrafast event during the captures (multi-shot imaging), lacking the capability of imaging the luminescent transient events, monochrome scaled captures, low number of captured frames (short duration of recording), demanding storage and transmission capacity requirements, extremely high built costs, high maintenance, oversized dimensions and highly complex operations. Coded compressive rotating mirror (CCRM) camera^6^ is a low-cost and compact novel high-speed imaging system that enables the capture of dynamic transient events in passive imaging mode in color format using optical encoding and compression hence facilitating the capture of events for longer durations with a considerably lower transmission and storage capacity requirements.

Within the wide range of high-speed imaging applications and alongside the requirements such as low build costs, compact dimensions and easy-to-operate functionality, there are some particular areas that demand several other key properties from their imaging systems such as the highly secured and encrypted data with compressed formats. Some of these applications include under-vehicle inspection^7,8^, quantum-secured imaging^9^, secured data storage and transmission using digital holography^10^, biometrics^11,12^ and military based applications^13,14^. Therefore by the advancements in the aforementioned fields, the requirement for fast and secured data encryption and compression becomes increasingly important.

There are several industry-standard methods^15,16^ such as advanced encryption standard (AES)^17^ and Rivest-Shamir-Adleman (RSA)^18^ that have been widely adapted in industrial applications and have also been used within the current computer operating systems. In addition to these techniques, other encryption methods that has also been used in imaging fields can be divided into several categories^19^ of chaos^20–25^, DNA based^26–28^, cellular automata^29–31^, fuzzy logic^32–34^ and transform based (e.g., Wavelet, Fourier, Frensel etc)^35–38^ as well as cryptographic techniques using compressive sensing (CS)^39–41^ in digital domain. These techniques require the initial acquisition and storage of the data in the memory of a processing unit where the aforementioned techniques can access the raw data and apply the encryption in a separate step. Therefore, the original and unsecured data require a large amount storage and transmission capacities, lengthy processing times and can be easily attacked by the intruders while in their unencrypted format.

Furthermore, optical based methods have been widely utilized in the field of image cryptography that is due to their faster computational speed and data processing in different optical domains. Methods such as double random phase encoding^42^ that implements two random phase diffusers in space and frequency domains, optical colour image encryption^43^ and multi-beams interference with vector composition^44^, utilize the advantages of optical imaging encoding where the data are stored in their encrypted format. These methods can be applied on low-speed imaging systems where the data acquisition rate is the same as the frame rate of the detector. Additionally, these types of image encryption techniques only apply the optical encryption on the original data where the compression is an extra step that is commonly applied on the data after they have been stored on a memory unit hence requiring high storage and transmission capacities, similar to those methods in the digital domain. Therefore achieving both data encryption with high security and high compression in the optical domain, becomes a necessity to overcome the aforementioned shortcomings on the conventional data encryption-compression modalities.

To address these matters, we utilize the CCRM camera^6^ to securely capture and compress the dynamic scene with high frame rates entirely in optical domain, hence overcoming the limitations of aforementioned video encryption and compression techniques. CCRM camera captures transient events in passive imaging mode and by relying on its optical encoding (key) and continuous frame sweep on the detector surface that achieves the highest compression ratio of 368 and highest sequence depth of 1400 frames form a single exposure (single capture) of the detector. The heavily compressed data from the CCRM camera that are encoded by the optical mask can only be decrypted using the observed pattern on the detector which is considered as the “key” to the reconstruction process. Alongside the high capture rate of the CCRM camera, the highly secured and compressed data format eliminates the aforementioned drawbacks of the conventional digital and optical encryption-compression methods and provides a new imaging platform for the applications such as medical and military based imaging systems where the confidentiality of the data remain the first priority.

## Operation of CCRM camera

**Figure 1:**
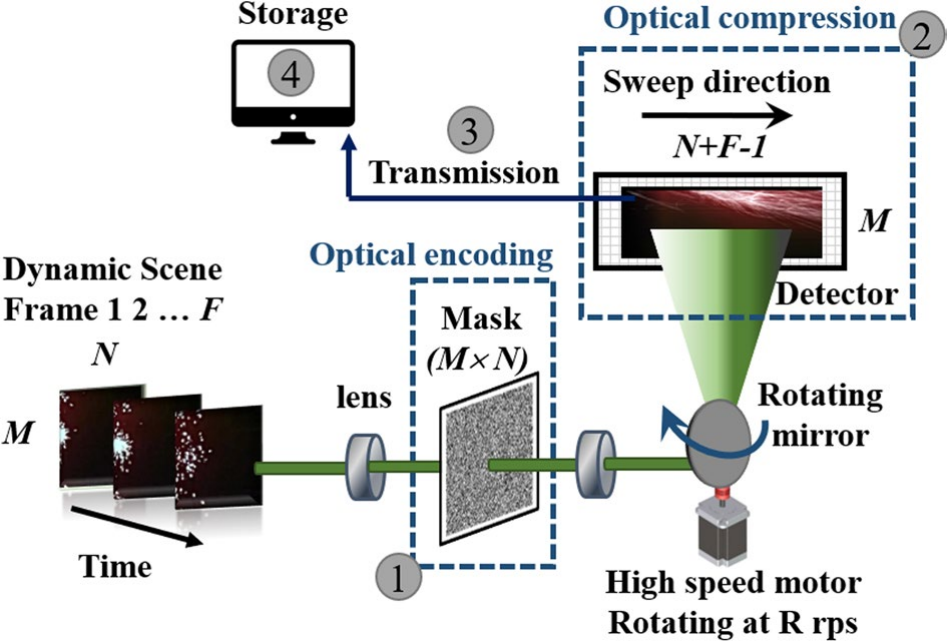
Configuration and operation principle of the proposed CCRM camera setup.

Depicted in Fig. 1 is the configuration and operation principle of the coded compressive rotating mirror (CCRM) imaging system. During the capture of a dynamic scene, the image at time t is focused on an static optical mask (noted as 1) that is printed on a soda-lime glass and consists of a random binary pattern with 50$\%$ transmission ratio. The optically encoded image is then focused on the mirror rotating at R (rps) by a high speed motor and reflected towards a 2 dimensional (2D) detector (such as Complementary metal-oxide-semiconductor (CMOS) or charge-coupled device (CCD)). The rotation of this mirror sweeps the individual frames across the surface of the detector module based on their time of arrival and overlaps them (optical compression) during a single exposure that creates a single pixel shift in-between the adjacent frames (noted as 2). Capturing a scene in a single exposure of the detector, eliminates the limitation of digitization and readout time of the camera from the proposed scheme. The captured encoded and heavily compressed 2D frame is then transmitted through a channel and stored on a storage unit.

The mathematical representation of data acquisition process of the proposed CCRM imaging system can be formulated as

1 $$\begin{aligned} y = TCAx + n, \end{aligned}$$

where $y \in {\mathbb {R}}^{MN+(F-1)M \times 1}$ is the captured data by the detector in a vectorized format, $T \in {\mathbb {R}}^{MN+(F-1)M \times MNF}$ is the linear operator of frame shifting and overlapping that is built upon F identity matrices, $C \in {\mathbb {R}}^{MNF \times MNF}$ is the obtained motion profile of the sweep from the calibration points on the encoder in the form of a diagonal matrix, $A \in {\mathbb {R}}^{MNF \times MNF}$ is the matrix that holds F encoding pattern of $M \times N$ in a diagonal form, $x \in {\mathbb {R}}^{MNF \times 1}$ represents the original frames in a vectorized format, and *n* is the additive zero mean Gaussian noise.

y represents the spatially coded and compressed observed data on the detector that contains the aggregate of individually encoded and temporally overlapped frames where each frame is positioned with a single pixel shift in the horizontal axis (sweep direction) compared to its adjacent frames. All the depicted operations in Eq. (1) occurs in the optical domain and therefore the entire function of encryption and compression happen at the operation speed of the imaging system without any requirements for external processing power or subsidiary operations at the digital domain. Here, we implement the Alternating Direction Method of Multipliers (ADMM)^45^ using the total variation (TV)^46^ as the regularizer function to decrypt and decompress the scene (${\hat{x}}$) from observed data (y).

This approach transforms the obtained equation into a minimization problem and solves the equation by minimizing the energy function via iterative calculations.

Decryption and decompression of the original scene can be achieved by solving the minimization problem that is

2 $$\begin{aligned} {\hat{x}}= \arg \min _x{\frac{1}{2}\left| \left| y-TCAx\right| \right| _2^2+\rho _kD\left( x,\rho _{tv},\ w_{tv}\right) } \end{aligned}$$

where $\rho _k\ ,\ \rho _{tv}$ are the variable regularization and denoiser threshold parameters that are adjusted based on the calculated error at each iteration and $w_{tv}$ is the regularizer weight for each horizontal, vertical and temporal domains and *D* is a regularization function that promotes sparsity in the dynamic scene. The linear operation of frame shifting and overlapping (represented by operator T) results in the compression and encryption of the data in CCRM camera^4^.

## Encryption properties of CCRM camera

Optical encoding using binary encoder patterns have been previously utilized in compressive sensing (CS) based high-speed imaging technologies^1–4,47^ where they have been considered as encoding patterns that enabled the temporal compression and the reconstruction of the data. This encoding pattern however has another significant role in high-speed imaging applications that is to efficiently encrypt the data in the optical domain which could have a high impact in the security of the captured data^48^. The conventional data encoding techniques require all the raw data to be stored in an accessible storage unit prior to going through the encryption or compression stages. This process required a considerable amounts of storage and transmission capacities and in the cases of digital encryption methods - leaving the confidential data exposed to the possible threats. The sequential operations of optical encoding and compression in the CCRM camera enables the real-time data encryption and eliminates the potential exposure of the data.

**Figure 2:**
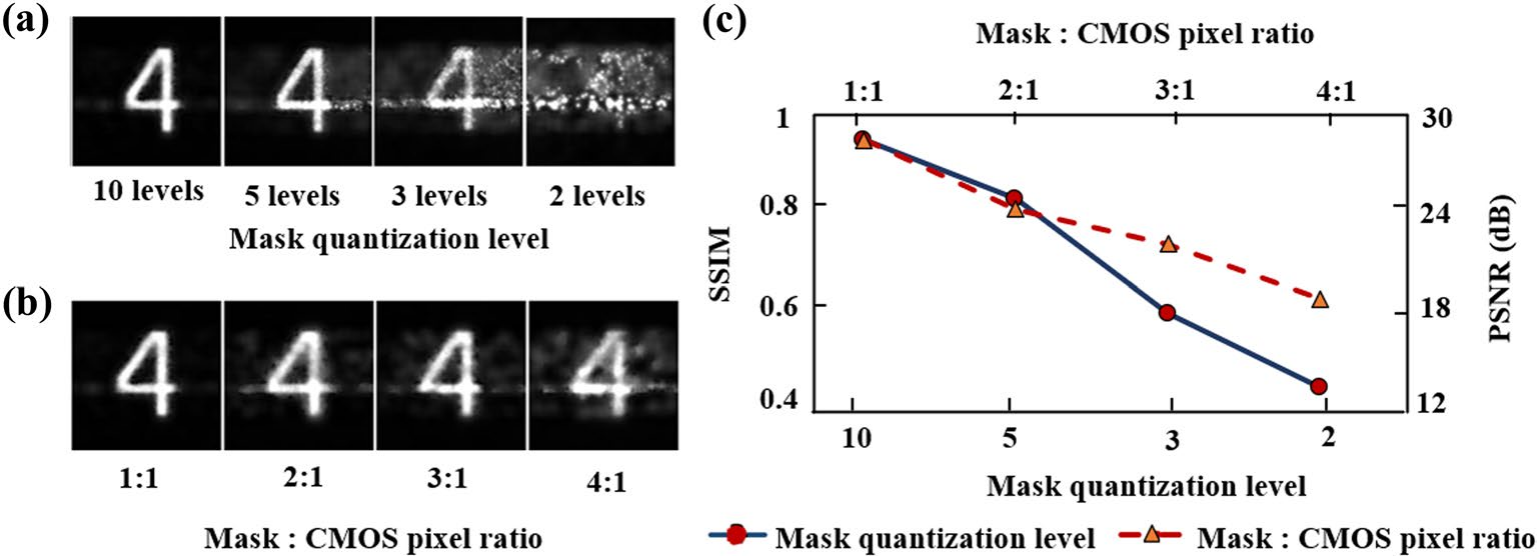
Reconstructed data (**a**) using various mask patterns quantised at different levels (**b**) at various Mask to CMOS pixel ratio. (**c**) SSIM and PSNR measurements for the figures shown at section (**a**, **b**).

In the optical setup of the CCRM camera, as the light transmits through the optical mask, the intensity values of the adjacent pixels interfere with the neighbouring pixels due to the light diffraction and changes the encoder pattern from binary into grey scale pattern. In the previous studies^2,48^ techniques to reverse the encoder pattern back into the binary format has been performed by implementing the commonly used binning process (e.g., 2 $\times$ 2 or 3 $\times$ 3) on the detector however this comes at a cost of the reduced spatial resolution of the detected image. CCRM camera exploits this feature of the optical encoder where by attaining a reference image of the encoder pattern on the detector prior to capturing the dynamic scene, the requirements for the binning process is eliminated therefore achieving higher spatial resolution compared to the aforementioned methods. Here, we configure the CCRM camera to capture a scene from the U.S. Air Force (USAF) static target (G2-E4). As the dynamics of the scene in the frames are constant over time, by taking a single image of the target a reference image representing all the frames in the scene is obtained. This reference image is then used to evaluate the performance of the reconstruction algorithm by calculating the Peak Signal to Noise Ratio (PSNR) values and the Structural SIMilarity index measurement (SSIM)^3,4^. Figure 2 shows the effect of quantizing the observed encoder data on the detector where it shown that the data reconstruction using the observed greyscale pattern has the highest reconstruction quality. Furthermore, increasing the dimension of encoder pixels (increased ratio between mask and detector pixel size) also reduces the reconstruction quality of the data when there binning process is not applied on the detector. We perform analysis on four datasets of blood cells flowing in a microfluidic chip (experimental data - courtesy of Kim Ulvberget @Green-life.no)^49^, droplet stream (experimental data from CCRM camera), speaking person (synthetic video dataset)^50^ and lighter spark (experimental data from CCRM camera). The authors confirm that all the procedures were performed in accordance with the relevant guidelines and regulations.

These datasets represent different types of applications and have diverse characteristics such as the object movement speed and direction, dynamic range, spatial resolution etc.

**Figure 3:**
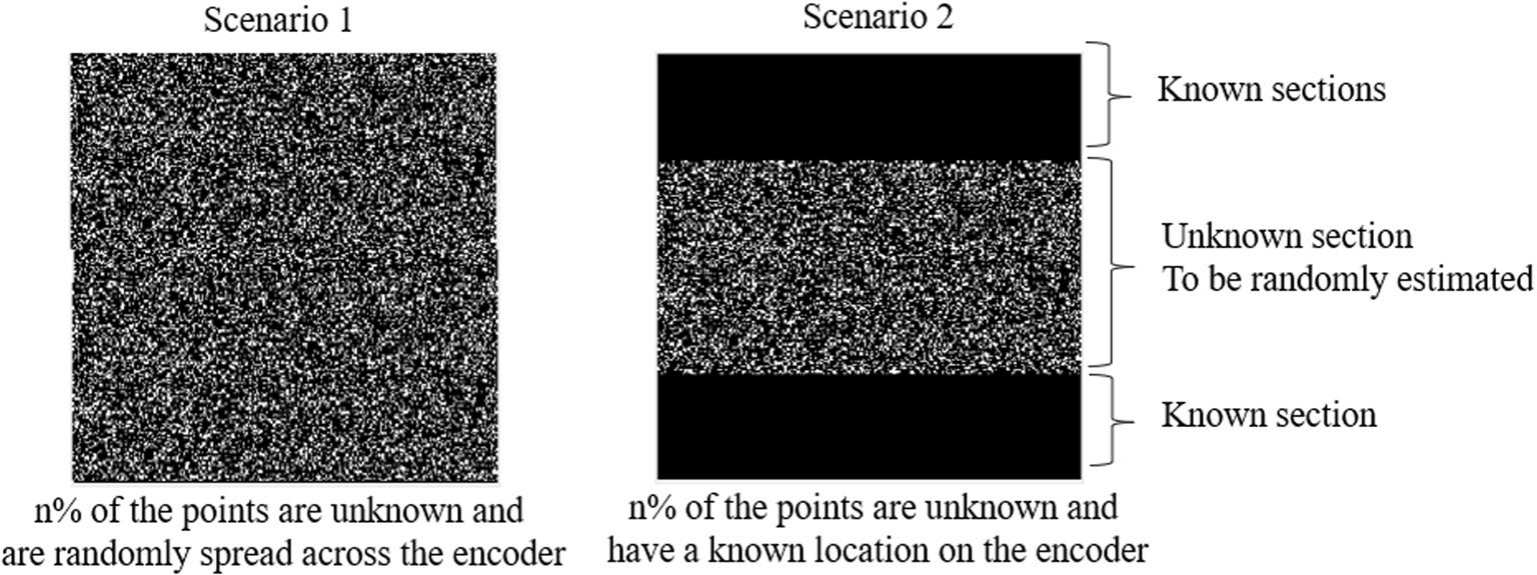
Example of the absolute difference between the original and the estimated encoder patterns at 50$\%$ data validity for the two presented scenario.

Key Analysis (KA) is often considered as a fundamental part of any encryption method that plays a critical role in defining the strength of the algorithm. Strong secret keys will have a large space and high sensitivity^51^. Key space corresponds to the overall dimensions of the key where larger dimensions decrease the overall probability of estimating the secret key. Key sensitivity on the other hand, relates to the decryption using a partially known information from the secret key. Assuming the case where the decoder has no information about the secret key, given the number of k = 122,500 elements in the 2D encoder A($350\times 350$), the total number of possible keys is ${10}^{122,500}$ hence the probability of having the exact encoder pattern is estimated as $P\left( A\right) \ =\ 1/{10}^{122,500}$ which could be considered as an infinity small number. Base 10 in this expression is due to the fact that we use grey scaled pattern that is observed at the detector for reconstruction of the data. For the key sensitivity analysis, we use Mean Squared Error (MSE) and SSIM as the two main parameters for reconstruction quality assessments for the scenario that one has partial knowledge about the secret key. In the first scenario, we assume that the known key values to an unauthorised person with various known percentages are evenly spread across the encoder matrix. Therefore, in this scenario there is no continuity in the pattern of the known elements of the encoder. The second scenario however, considers that the partial yet continues sections of the encoder with various known percentages are known to an unauthorised person. In this scenario, we assume that the missing information from the encoder are located at the centre of the encoder matrix where they will be randomly estimated and the top and bottom section of the matrix hold the true encoding data. Depicted in Fig. 3 are the absolute difference between the true encoding pattern and the predicted pattern.

The black sections in figure represent the known information and the grey pixels show the differences between the original and the predicted values respectively. As the original encoder is assumed to be a grey scale pattern with 10 different intensity levels, the brighter pixels represent higher differences between the original and the estimated pixels.

**Figure 4:**
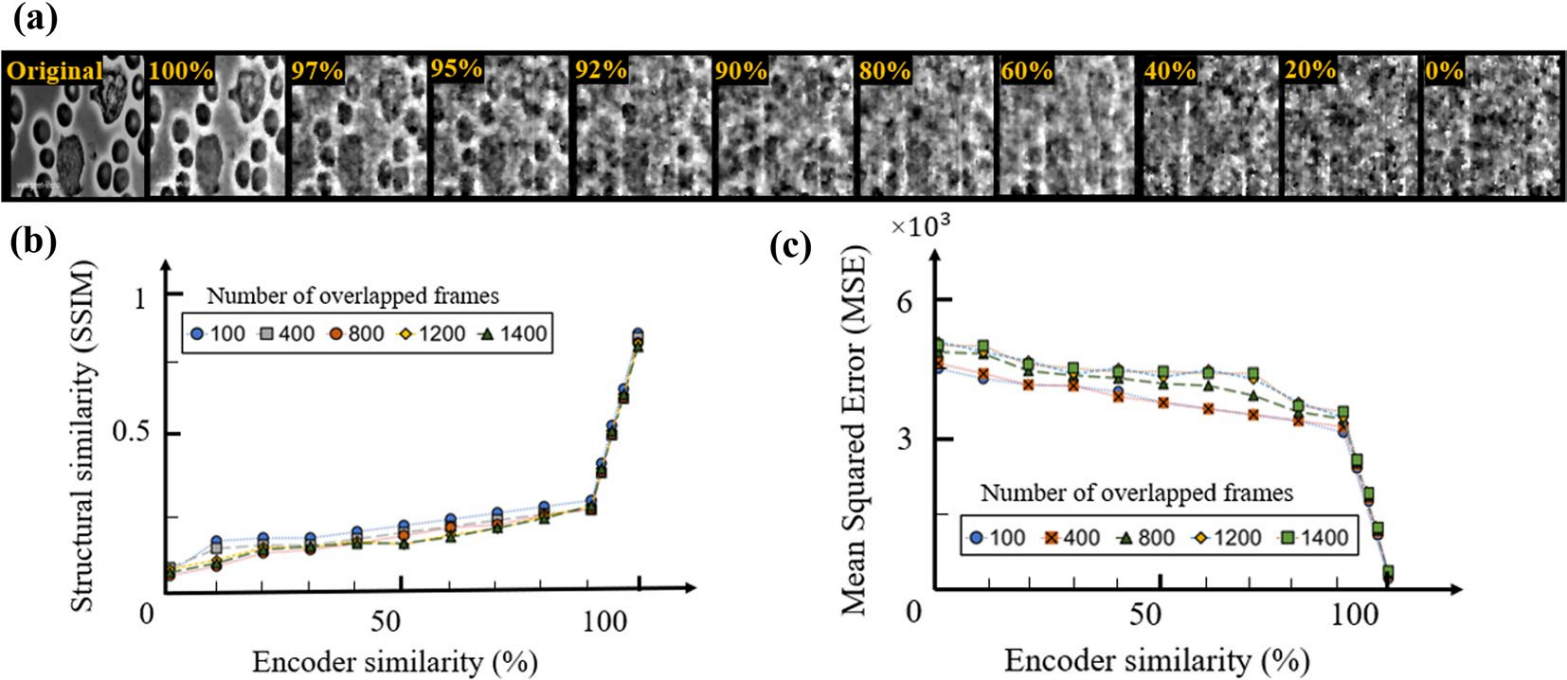
(**a**) Reconstructed frames, (**b**) SSIM and (**c**) MSE measurements of the immune cell dataset for the partially known encoder data at various known percentages of the true values located at random pixel positions.

Depicted in Fig. 4 are the reconstructed frames (for the case of 100 overlapped frames), SSIM and MSE measurements from immune cells dataset at various known percentages for scenario 1 and it shows that reconstruction quality of data is extremely sensitive to small percentage of unknown pattern on the estimated encoder.

**Figure 5:**
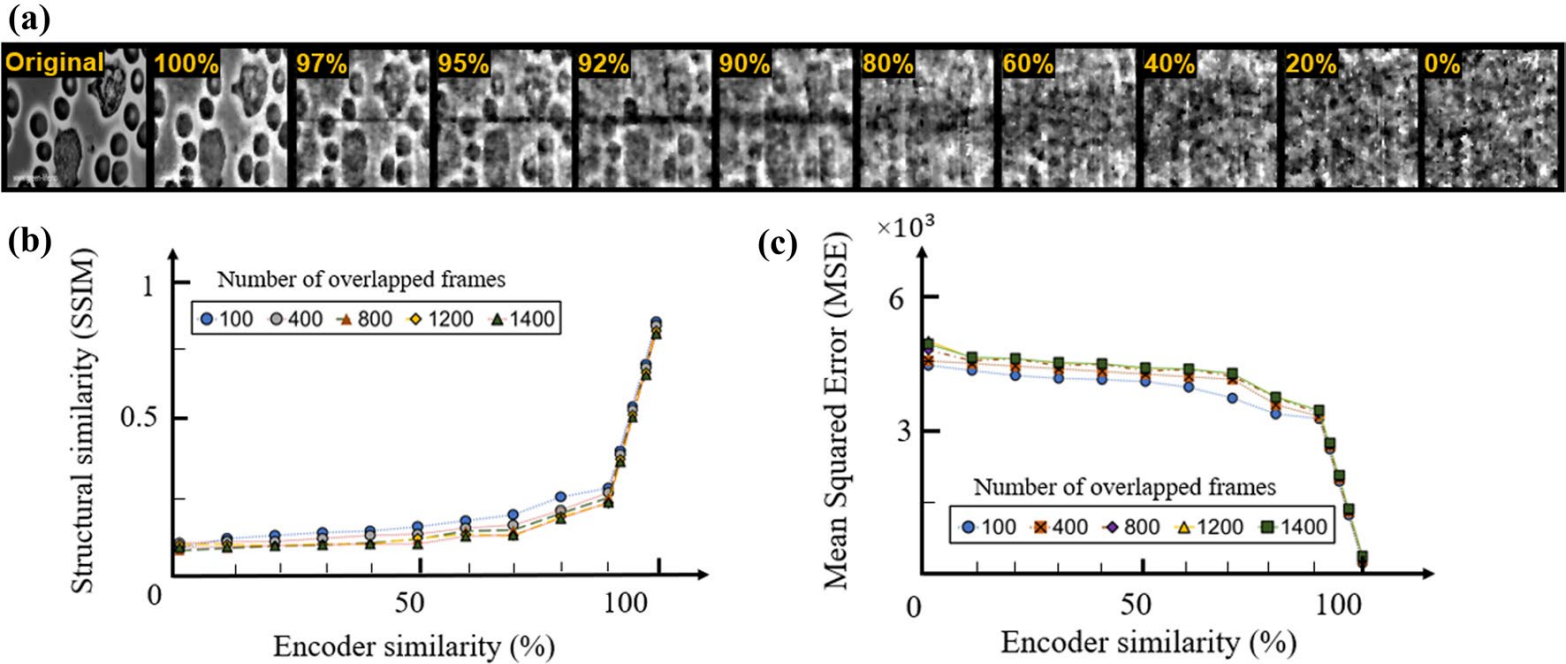
(**a**) Reconstructed frames, (**b**) SSIM and (**c**) MSE measurements of the immune cell dataset for the partially known encoder data at various known percentages of the true values with the unknown key values located at the central section of the encoder.

The same types of recovery curves were observed on the same dataset in scenario 2 (depicted in Fig. 5) and three other aforementioned datasets (figures are not included due to the similarities in the graphs). Based on these presented data, with 95$\%$ correctness of the encoder values the SSIM is reduced to  0.5 from 0.85 at 100$\%$ similarity and the MSE is increased from 70 to  2200. This can be viewed as the minimum acceptable percentage for the encoder to achieve an acceptable data recovery in these datasets.

**Figure 6:**
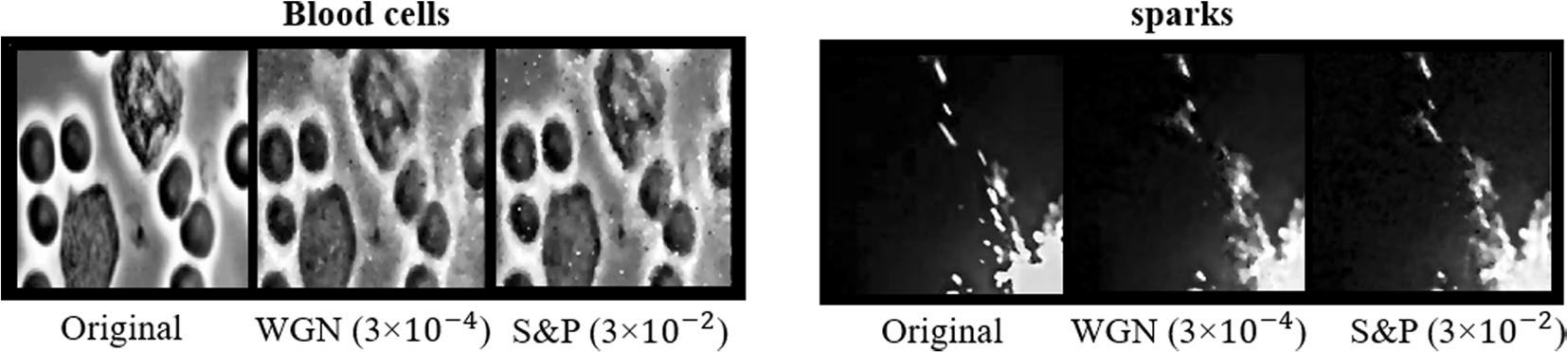
Extracted frames from decrypted video sets at various noise levels for additive Gaussian noise and salt and pepper noise.

**Figure 7:**
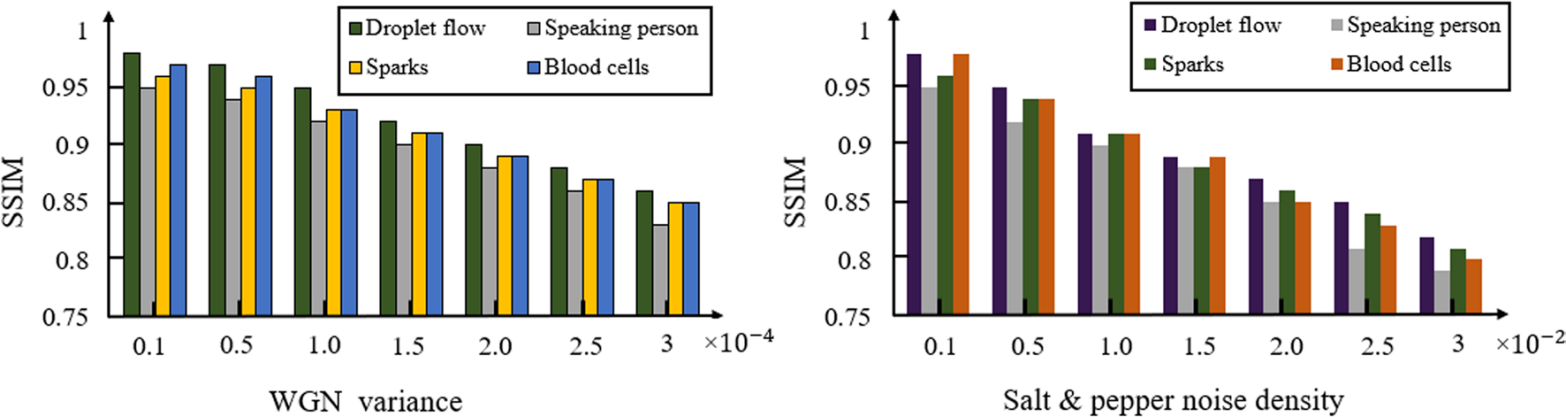
SSIM values of decrypted data at various noise levels for additive Gaussian noise and salt and pepper noise.

During the transmission stage over a channel, data can be altered by a variety of interference and additive noise where a robust decryption algorithm can recover the frames with low data loss^23,38^. To test the performance of our decryption algorithm, two types of Salt and pepper noise (SPN) and additive white Gaussian noise (AWGN) are added to the encrypted data. Depicted in Figs. 6 and 7 are extracted frames and SSIM values from decryption of data at various noise levels respectively where due to the nature of the proposed reconstruction algorithm (iterative denoising defined as *D*(.) in Eq. 2), a robust and low loss data reconstruction is achieved.

Information Entropy (IE) measures the uncertainty of the information occurrence per bit in an image and is widely used in the applications of image compression and encryption^23,38^. Shannon’s source coding theorem describes the definition of the optimal coding by the length of the code assigned to the i’th symbol (pixel) that is $-log_2P(i)$ where *P*(*i*) is the probability of the occurrence of the symbol i. The entropy H(p) is calculate as:

3 $$\begin{aligned} H(p)= -\sum \limits _{i=1}^n P_i \times log_2P(i) \end{aligned}$$

that is measured in bits per symbol (pixel values) where n is the number of the possible values per pixel. Assuming an 8 bit image, the ideal encryption method will have the entropy of 8. The mean calculated entropy of the aforementioned datasets is 7.48 with the min and max values of 7.34 and 7.64 that are very close to the expected ideal encryption value. Depicted in Table 1 are the full list of entropy measurements for various compressed and encoded frames for the aforementioned datasets.

**Table 1:** Information entropy of encrypted images at various compression rates.

Data set	Number of frames								Mean	Max	Min
	100	200	400	600	800	1000	1200	1400			
Blood cells	7.34	7.62	7.64	7.56	7.49	7.43	7.36	7.40	7.48	7.64	7.34
Droplet flow	7.51	7.65	7.45	7.51	7.41	7.40	7.35	7.41	7.46	7.65	7.35
Speaking person	7.45	7.55	7.52	7.52	7.48	7.45	7.39	7.49	7.48	7.55	7.39
Sparks	7.54	7.54	7.42	7.49	7.45	7.44	7.40	7.52	7.47	7.54	7.40

Furthermore, Correlation Coefficient (CC)^51^ is another important analytical factor which measures the similarity between the corresponding pixels of an original and the reconstructed image that is obtained by the following equation

4 $$\begin{aligned} C C=\frac{\sum _{m} \sum _{n}\left( A_{m n}-{\bar{A}}\right) \left( B_{m n}-{\bar{B}}\right) }{\sqrt{\left( \sum _{m} \sum _{n}\left( A_{m n}-{\bar{A}}\right) ^{2}\right) \left( \sum _{m} \sum _{n}\left( B_{m n}-{\bar{B}}\right) ^{2}\right) }} \end{aligned}$$

**Figure 8:**
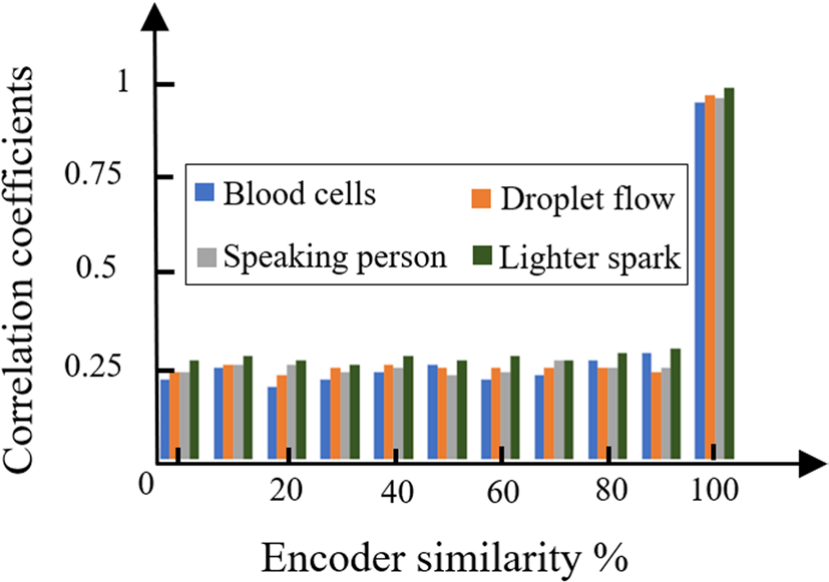
Calculated correlation coefficients (CC) of reconstructed data using encoder patters with various similarity percentages to the original encoder key.

CC is the correlation coefficient between the plain and the reconstructed image, A is the plain image, B is the reconstructed image, ${\bar{A}}$ is the mean of the plain image, ${\bar{B}}$ is the mean of the reconstructed image and m, n are 2D dimensions of the images. Lower amounts of correlation indicate stronger encryption strength when the data are reconstructed with partially known encoder information. The mean calculated CC measurement for the reconstructed data with encoder similarity of 0-90$\%$ in the aforementioned datasets are 0.25 with a minimum and maximum values of 0.22 and 0.28 respectively and therefore it is evident that the observed data from the detector are efficiently secured and are very sensitive to the slightest changes to the encoder data. Depicted in Fig. 8 is the measured CC values of the reconstructed data for different datasets at various encoder similarity percentages. Randomly estimated sections in the encoder effect the reconstruction quality of the data and to estimate this deviation in the reconstruction fidelity, the process is repeated 50 times where during each trial, a new randomly estimated encoder is used for data reconstruction.

In the industry-standard encryption techniques such as AES^17^ and RSA^18^ methods, the decryption stages also show high sensitivity to the changes in the keys where in the AES method, changing a single byte of the key prevents the decryption algorithm from recovering the data where the CC value drops to less than 0.1. RSA algorithm is also sensitive to the private key where by randomly estimating the private key an average CC of 0.23 is obtained from the decrypted data.

CC analysis can be further extended and applied on every pair of adjacent pixels in the encrypted frames where encryption schemes are expected to hide such correlations among pixels^23,24,38^. The obtain this inner-frame measurement, correlation values of adjacent pixels in three directions of horizontal, vertical and diagonal are calculated against the selected pixel and the correlation among the pixel pairs can be calculated as

5 $$\begin{aligned} & r_{{xy}} = \frac{{(MN)^{2} \cdot \text{cov} (x,y)}}{{\sum\nolimits_{{i = 1}}^{{MN}} {\left( {x_{i} - E_{x} } \right)^{2} } \cdot \sum\nolimits_{{i = 1}}^{{MN}} {\left( {y_{i} - E_{y} } \right)^{2} } }} \\ & E_{x} = \frac{{\sum\nolimits_{{i = 1}}^{{MN}} {x_{i} } }}{{MN}} \\ & \text{cov} (x,y) = E\left( {\left( {x - E_{x} } \right)\left( {y - E_{y} } \right)} \right) \\ \end{aligned}$$

where (x, y) is the combination of two directions (horizontal, vertical or diagonal) for the adjacent pixel pair. To measure the correlation of adjacent pixels, we select and analyse 1000 pairs of adjacent pixels from random locations of the original and encrypted images. Shown in Table 2 are correlation and correlation coefficients calculated from Eq. (5) where it shows low correlation between the adjacent pixels hence the effectiveness of the proposed method against statistical attacks.

**Table 2:** Correlation analysis of adjacent pixels in horizontal, vertical and diagonal directions.

Blood cells flow			Droplet stream			Speaking person			Spark		
H	V	D	H	V	D	H	V	D	H	V	D
0.982	0.925	0.963	0.955	0.924	0.934	0.941	0.975	0.910	0.941	0.932	0.910
0.021	0.015	0.018	0.019	0.017	0.019	0.019	0.018	0.016	0.019	0.017	0.021

In addition to the CC measurements, the Execution time (ET) is another critical parameter that is the time required to execute a given image or video encryption-compression process which is typically considered as the combination of the compile and the run time of the algorithm in the digital domain. For practical implementation of image encryption, ET must be minimum for given data size. In the conventional schemes that are dependent on algorithms in the digital domain, this process can take between sub-second to seconds per frame ($500 \times 500$ in RGB) and is linearly proportional to the dimensions and number of the frames, e.g. it takes   0.25 s per frame in^51^ that is more than   5.8 min to encode 1400 frames of a video. Industry standard AES and RSA methods are capable of encoding at the rates of 100 Mbps and 1 GBps respectively using a standard i5 2.5 Ghz central processing unit (CPU). Hence encryption of 1400 frames with the aforementioned dimensions takes   1.4 min using the AES-128 encryption and   7 s for the RSA method whereas in the proposed CCRM scheme, the joint operations of encryption and compression takes 12 ms that is significantly faster than the conventional methods. This time is dependent on the rotation speed of the motor and the size of detector, and is independent of the dimension and number of frames.

Recording the dynamics of a phenomena often require multiple captures at different points in time (lifetime-based screening and characterization of fluorescent proteins, microfluidics analysis etc) or several captures from various viewing angles (textile strength testing, combustion and chemical reactions etc) depending on the nature of experiments. Therefore, the required storage and transmission capacities increase drastically and therefore data compression methods are often employed in such scenarios.

Compression is regarded as a reversable conversion of data that contains fewer number of bits compared to the original format which facilitates a more efficient storage and transmission of data. Data compression can be divided into two types: lossless and lossy techniques. Lossless compression is predominantly used for text or application files where a loss of information even at a very low rate can cause a major damage to the data. Lossless compression methods often use statistical information to reduce the data redundancies.

Huffman-Coding^52^ and Run Length Encoding^53^ are two common algorithms that allow for compression ratios of  2:1^54^. On the other hand, lossy compression introduces some errors to the data during the compression stage and yet can be used for data types such as images, video and sound which contain large amounts of redundant data. These methods are capable of achieving compression at the rates of up to 10 Mbps using the aforementioned processing unit hence the joint operation of compression and encryption for the RGB video with dimension of $500\times 500 \times 1400$ will take   15 min to complete. In these methods however high amounts of compression ratios often result in lower decompression quality that is seen as a trade-off in lossy compression methods.

The compression process typically takes place prior to the encryption stage as the compression utilizes the sparsity in the spatial and temporal domains (intra-frame and inter-frame compression) in the data. H.26(1,3,4,5) and MPEG-(1,2,4)^55,56^ are two of the commonly used lossy compression methods for video data where compression ratios of 200:1^55,56^ can be obtained without losing substantial amounts of information from the frames. These methods however are associated with a drawback that is by having higher amounts of compression, the quality of the de-compressed data is linearly reduced [50]. As an example, compression ratios of 50:1 and 200:1 yield SSIM of 0.95 and 0.84 respectively that will continue to decrease with higher compression ratios.

**Figure 9:**
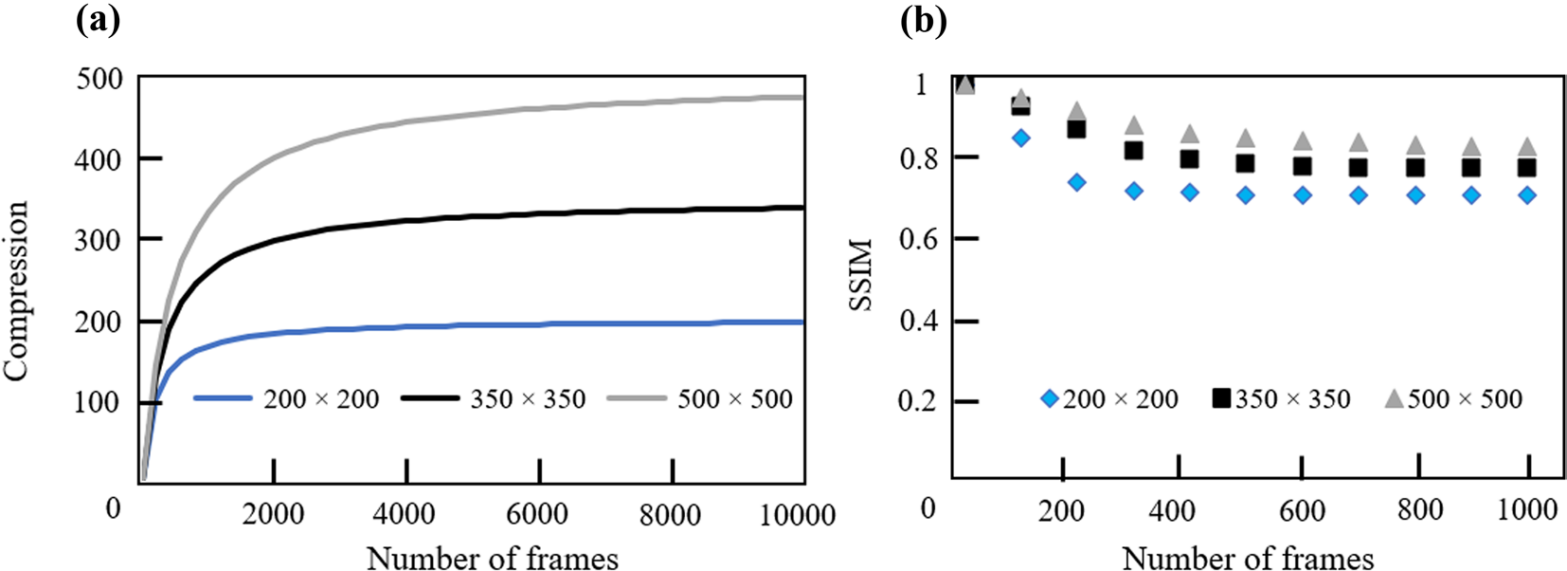
Graphs of the (**a**) compression ratios and (**b**) SSIM measurements against number of frames in CCRM camera.

As it is shown in Fig. 9, the compression ratio and SSIM measurements are independent of each other in the CCRM camera where they only depend on the spatial dimensions of the scene where the SSIM measurements tend to converge to its lowest value at number of frames >= 2 times of the dimension of the frame. This enables the continuous recording and compression of the data without sacrificing the data quality in the reconstruction process. CCRM camera achieves high compression ratios (333 and 476 for 1k and 10k number of frames respectively) and maintains high decompression (reconstruction) quality by attaining high SSIM values (higher than 0.8 for 10k frames).

Variance analysis^57^ is another measurement to test the performance of the data encryption technique. Closeness of the variance between the encrypted data shows the strength of the encryption algorithm while the encoder (key) varies. Variance histogram analysis is defined as

6 $$\begin{aligned} V A R(Z)=\left( \frac{1}{MN}\right) \sum _{i=1}^{M} \sum _{j=1}^{N}\left( \frac{1}{2}\right) \times \left( z_{i}-z_{j}\right) ^{2} \end{aligned}$$

where Z is the vector of the histogram values and Z = $z_1,z_2,\ldots ,z_2 56$, $z_i$ and $z_j$ denote the number of pixels respectively.

**Figure 10:**
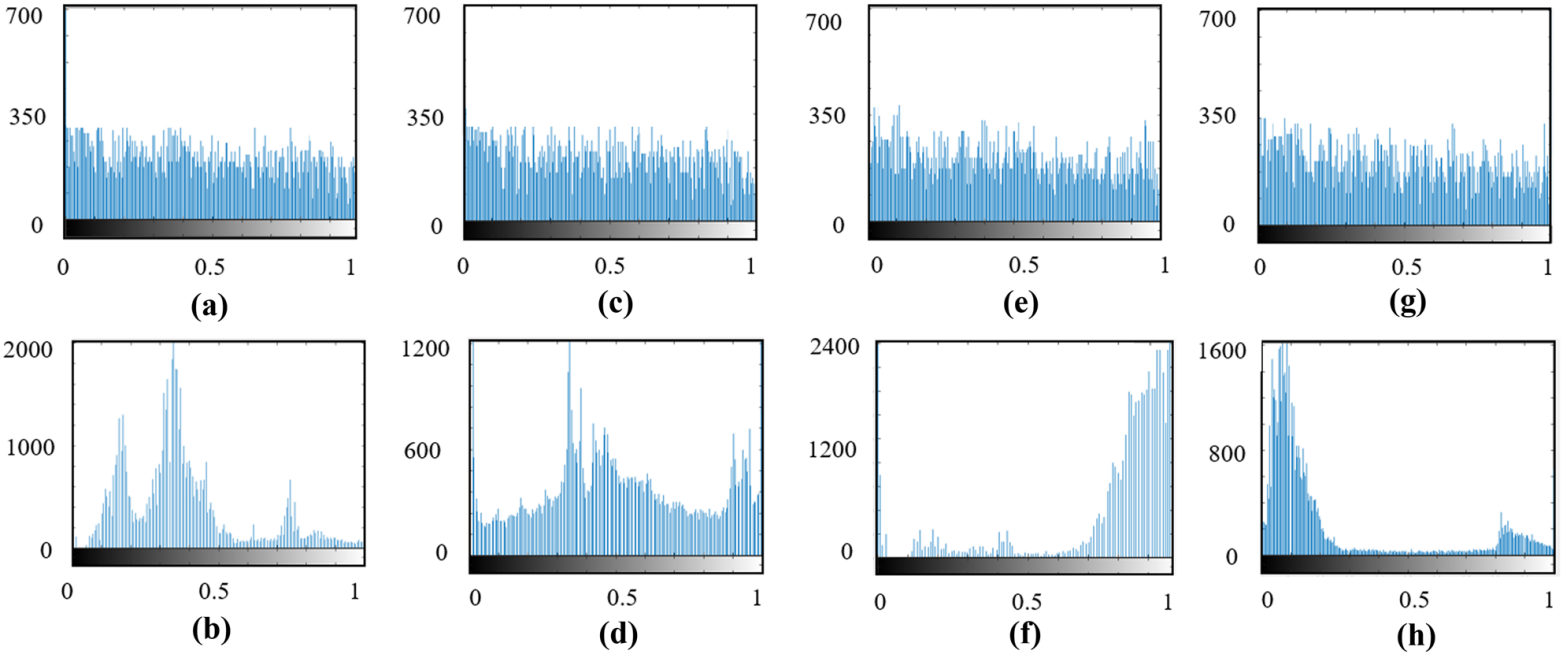
Histograms of the (**a**, **b**) speaking person, (**c**, **d**) blood cells, (**e**, **f**) droplet flow and (**g**, **h**) sparks data sets for encrypted and compressed formats (top row) and a single frame from data set (bottom row).

Assuming *A* to be the key that is used to encrypt the frames, variance analysis can be conducted by by slightly changing the original key value and performing the encryption on the same set of frames. In the proposed scheme, as the key is represented by a 2-dimensional matrix, we change the matrix (key) values at increments of 20% and calculate the variance of the histogram. Depicted in Fig. 10 are the histograms of the encrypted and compressed data sets (top row) and histograms of single frames from data set (bottom row) respectively.

Table 3 shows the calculated variance values for various encoding keys with difference percentages to the original key and it is evident that changes in the calculated variance values are small (encrypted images are uniform).

**Table 3:** Variance analysis.

Dataset	0%	20%	40%	60%	80%	100%
Blood cells flow	350.553	348.293	396.456	356.660	324.210	339.550
Droplet stream	340.226	372.930	366.655	355.623	350.612	370.480
Speaking person	380.570	360.589	362.569	378.569	381.010	377.960
Spark	330.850	342.896	358.560	349.810	365.650	341.812

The proposed CCRM camera overcomes these fundamental limitations where it can achieve both compression and encryption at ultra-high rates (12 ms) through the native built-in optical operation in which the requirement for storage and transmission capacities are substantially reduced. The optical encoding mechanism in CCRM camera, enables a secure data storage and handling in the imaging applications fields (e.g. medical imaging and military based applications) where information security and confidentiality remains one of the top priorities.

## Conclusion

In this paper, we demonstrated the encryption and compression properties of the CCRM camera where it has shown to be a formidable imaging system for applications that demand highly encrypted and compressed data acquisition at high frame rates in a compact design and easy-to-use operation. CCRM camera integrates the video encryption and compression in the optical domain hence significantly improving the information security, storage and transmission capacities as well as achieving the highest compression ratio of 368 and the highest sequence depth of 1400 reconstructed frames from a single shot image acquisition compared to the other CS based imaging techniques. Conducted experiments demonstrate that the original data can only be recovered using the encryption key observed by the detector. Moreover by introducing amplitude encoding technique to the encryption and compression stages, the key-space has been significantly extended hence substantially reducing the risk of brute force attacks on the data recovery. CCRM camera can be implemented in a variety of applications such as medical and military based imaging systems where the data security alongside the storage and transmission capacities are considered as critical factors.
